# Research on the Mechanism and Process of Water-Jet-Guided Laser Annular Cutting for Hole Making in Inconel 718

**DOI:** 10.3390/mi16101090

**Published:** 2025-09-26

**Authors:** Qian Liu, Guoyong Zhao, Yugang Zhao, Shuo Yu, Guiguan Zhang

**Affiliations:** 1 School of Electrical and Electronic Engineering, Shandong University of Technology, Zibo 255000, China; 2 School of Mechanical Engineering, Shandong University of Technology, Zibo 255000, China

**Keywords:** water-jet guided laser, nickel-based alloys, film cooling hole, multi-pass annular cutting, processing mechanism

## Abstract

Nickel-based superalloys, serving as the preferred materials for hot-end structural components in aerospace engines, pose considerable challenges for the fabrication of high-quality gas film holes on their surfaces due to their inherent high hardness and strength. Water-jet-guided laser processing technology has exhibited notable potential in the realm of gas film hole fabrication; however, its engineering application is hindered by the lack of synergy between processing quality and efficiency. To tackle this issue, this study achieves efficient coupling between a 1064 nm high-power laser and a stable water jet, leveraging a multi-focal water–light coupling mode. Furthermore, an “inside-to-outside” multi-pass ring-cutting drilling strategy is introduced, and the controlled variable method is employed to investigate the influence of laser single-pulse energy, scanning speed, and pulse frequency on the surface morphology and geometric accuracy of micro-holes. Building upon this foundation, micro-holes fabricated using optimized process parameters are analyzed and validated using scanning electron microscopy and energy-dispersive spectroscopy. The findings reveal that single-pulse energy is a pivotal parameter for achieving micro-hole penetration. By moderately increasing the scanning speed and pulse frequency, melt deposition and thermal accumulation effects can be effectively mitigated, thereby enhancing the surface morphology and machining precision of micro-holes. Specifically, when the single-pulse energy is set at 0.8 mJ, the scanning speed at 25 mm/s, and the pulse frequency at 300 kHz, high-quality micro-holes with an entrance diameter of 820 μm and a taper angle of 0.32° can be fabricated in approximately 60 s. The micro-morphology and element distribution of the micro-holes affirm that water-jet-guided laser processing exhibits exceptional performance in minimizing recast layers, narrowing the heat-affected zone, and preserving the smoothness of the hole wall.

## 1. Introduction

As the modern aviation industry increasingly demands higher power from aeroengines, more stringent requirements have been placed on the service performance of hot-end structural components, including turbine blades, combustion chambers, and flame tubes [1,2,3]. Nickel-based superalloys, recognized for their outstanding creep resistance, fatigue resistance, and oxidation resistance, have become the preferred material for these hot-end components in aeroengines [4,5]. To enhance the stability and service life of hot-end structural components in aeroengines, it is essential to not only employ matrix materials with superior heat resistance and thermal barrier coatings but also to integrate advanced cooling technologies. Film cooling technology represents an effective external cooling method that generates a cooling film through numerous small holes (with diameters ranging from 0.2 to 0.8 mm) densely arranged on the surfaces of hot-end structural components. This technique effectively prevents direct contact between heat flow and component surfaces, thereby significantly improving the heat resistance of these components to meet the rigorous performance standards required by aeroengines operating in extreme high-temperature environments [6,7].

Nickel-based superalloys, distinguished by their high specific strength, low density, and exceptional stiffness, are critical materials that present significant machining challenges. During the micro-drilling process, these alloys often experience issues such as inlet and outlet defects, incomplete hole walls, and reduced dimensional accuracy [8]. In light of these challenges, the academic community has consistently directed its efforts toward achieving efficient and high-quality micro-drilling in nickel-based superalloys. Currently, the primary techniques for micro-hole machining in nickel-based superalloys include electrical discharge machining (EDM) [9], electrochemical machining (ECM) [10], and laser machining [11]. EDM is restricted to conductive materials and suffers from drawbacks such as low machining efficiency, considerable electrode wear, thick recast layers, and the formation of microcracks [12]. While ECM minimizes thermal damage during processing, its complex equipment requirements and cumbersome operational procedures have limited its widespread adoption [13]. In contrast, laser machining technology offers several advantages including high efficiency, excellent repeatability, and broad material compatibility. However, conventional long-pulse laser machining frequently leads to significant heat-affected zones and recast layers [14]. Although ultrafast pulse lasers theoretically facilitate “cold machining,” thereby circumventing heat-affected zones and recast layers altogether, their low efficiency coupled with high equipment costs renders them impractical for industrial applications [15]. Therefore, there exists an urgent need for an advanced manufacturing process that effectively balances quality with efficiency to surpass traditional methods.

Water-jet-guided laser technology constitutes an advanced composite processing technique that amalgamates conventional laser processing with water jet machining. This technology not only retains the merits of traditional laser processing, including high efficiency and versatility across a wide range of materials but also harnesses the cooling and impact effects of water jets to effectively diminish the thermal affected zone (TAZ) and eliminate molten slag, thereby pioneering new avenues for efficient and high-quality micro-hole processing [16,17]. Currently, scholars have delved into the interaction mechanisms between water-guided lasers and nickel-based superalloys from diverse perspectives [18,19,20,21,22,23,24]. Marimuthu [21] conducted a study on the fabrication of micro-holes on nickel-based superalloys coated with thermal barrier coatings (TBCs) using water-jet-guided laser, examining the mechanisms through which pulse frequency influences hole morphology characteristics under varying jet inclination angles. The findings revealed that an increase in pulse frequency results in smoother hole contours while mitigating heat-related defects such as coating delamination or recast layer formation. Cao [22] successfully fabricated micro-holes with a diameter of 0.7 mm and a wall roughness of less than 0.6 µm on nickel-based alloy specimens coated with TBCs through water-guided laser processing. The TAZ thickness was maintained below 1 µm, and no notable delamination or fracture was observed at the interface between the TBCs and the substrate, suggesting that water-jet-guided laser technology does not adversely affect the bonding strength between the coating and the substrate. Wang [23] constructed an energy coupling model between water-jet-guided laser and nickel-based alloys to elucidate the underlying mechanisms of the cooling effects associated with water jets. By optimizing the processing parameters and scanning trajectories in water-jet-guided laser machining, he effectively mitigated issues such as sharp-edge material fragmentation, coating delamination, and uneven sidewalls during hole drilling, ultimately achieving high-quality oblique holes with a recast layer thickness of merely 1.2 µm. Subasi [24] conducted drilling experiments on four aerospace nickel alloys using water-jet-guided laser technology to explore the influence of material thermophysical properties on machining removal efficiency, hole taper, and recast layer thickness. The research revealed a positive correlation between the material’s latent heat of vaporization and hole taper. Specifically, under the same combination of process parameters, nickel alloys with higher latent heat values exhibited relatively larger hole tapers, whereas those with lower latent heat values demonstrated smaller tapers.

In conclusion, the water-jet-guided laser processing technology exhibits notable advantages in the field of fabricating gas film cooling holes in nickel-based alloys. However, further exploration is still needed to achieve industrialized production of gas film holes with high precision, quality, and efficiency. Currently, the laser wavelengths predominantly utilized in water-jet-guided laser technology are 532 nm and 1064 nm. The 532 nm wavelength, characterized by its high energy density, low thermal damage properties, and extremely low water absorption coefficient, facilitates high-quality water–light coupling and holds a pivotal position in water-guided laser applications [25,26,27,28]. In contrast, the 1064 nm wavelength demonstrates a higher absorption coefficient in water compared to 532 nm. During the coupling process of high-power lasers with water beams, nonlinear effects such as optical breakdown and stimulated Raman scattering may emerge, potentially impacting the quality of water–light coupling and energy transmission. Nevertheless, the 1064 nm laser boasts remarkable stability, strong penetration capabilities, and favorable cost-effectiveness. Especially when processing materials like stainless steel, titanium alloys, and nickel alloys, its material removal rate stands out [29,30]. Therefore, conducting research on water-guided laser processing technology using the 1064 nm wavelength holds significant value for engineering applications.

In this study, we initially effectively diminished the peak power density at the water-laser coupling interface through the adoption of a multi-focal water–light coupling mode, successfully achieving efficient coupling between a high-power 1064 nm laser and a stable water jet, thereby elevating the initial laser power from 100 W to over 300 W [31,32]. Building upon this foundation, we systematically analyzed the material removal mechanisms in the water-jet-guided laser drilling of micro-holes in Inconel 718 nickel-based alloy. Furthermore, we conducted quantitative investigations into the influence patterns of key parameters, such as single-energy, scanning speed, and pulse frequency, on the geometric accuracy of micro-holes (diameter, roundness, and taper) in the context of the water-jet-guided laser ring-cutting drilling process. Subsequently, microscopic morphology characterization and energy spectrum analysis were performed on the micro-holes fabricated under optimized parameters. Ultimately, within the preferred process range, high-quality and high-efficiency micro-holes with an inlet diameter of approximately 820 µm and a taper angle of approximately 0.3° were successfully fabricated. The relevant findings provide a reliable process basis and experimental support for the subsequent application of water-jet-guided laser technology in the drilling of film cooling holes in Inconel 718.

## 2. Materials and Methods

### 2.1. Materials

In the experiment, Inconel 718 nickel-based superalloy with a thickness of 2 mm was utilized. Following forging, this alloy underwent solution treatment and aging heat treatment, resulting in a microstructure predominantly comprising a γ-phase matrix and δ-phase, with its chemical composition detailed in Table 1. Inconel 718 exhibits remarkable mechanical and fatigue properties in medium- to high-temperature environments, making it widely applicable in high-temperature components such as turbine rotor blades. The detailed mechanical properties of this alloy are outlined in Table 2. Prior to initiating the experiment, both sides of the specimens were abrasively polished using silicon carbide sandpaper to eliminate surface impurities and organic contaminants. Subsequently, the specimens were subjected to ultrasonic cleaning in anhydrous ethanol for a total of 15 min. Ultimately, the specimens were wiped clean with a lint-free cloth and allowed to dry naturally.

### 2.2. Experimental Equipment

**Figure 1:**
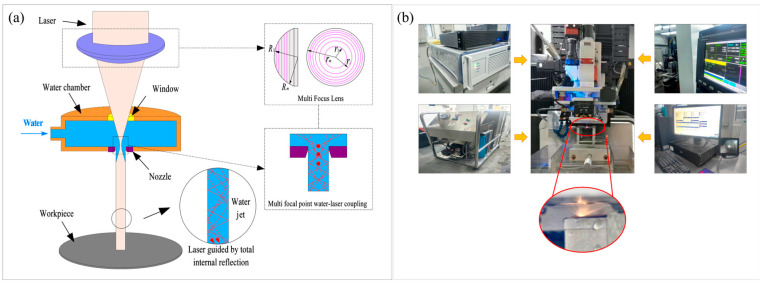
Principles and equipment schematic of water-jet-guided laser machining. (**a**) Principles schematic; (**b**) Equipment schematic.

This experiment utilizes self-developed water-jet-guided laser processing equipment, the working principle of which is illustrated in Figure 1. The equipment primarily consists of a laser light source, a high-pressure water supply system, a water–light coupling system, a precision displacement workbench, and a computer control system. The laser light source employs a Nanosecond fiber laser (YLPN-2-20 × 500-500, Amphoton, Changzhou, China) with a wavelength of 1064 nm, producing a Gaussian-distributed output beam. The pressure of the high-pressure water supply system can be adjusted within the range of 0~35 MPa. Deionized water is injected into the coupling water chamber through a hydraulic pump, generating a high-pressure water jet with a diameter ranging from 60 µm to 100 µm. The laser beam is precisely positioned by a CCD camera, achieving accurate coupling with the pure water jet within the coupling chamber. The computer control system enables the precision displacement workbench to move linearly or arcuately, facilitating complex processing trajectories.

To achieve water-jet-guided laser processing with high-power 1064 nm wavelength laser, a novel method of axial multi-focus point water–light coupling is proposed. This method utilizes a focusing lens with a multi-curvature structure to enable parallel beams to form multi-focus points on the mesoscopic scale along the beam axis. This significantly reduces the peak power density at each focusing point, preventing the laser peak power density in the water–light coupling region from exceeding the breakdown threshold of the aqueous medium, thereby enhancing the initial coupling power of the 1064 nm laser.

### 2.3. Methods

**Figure 2:**
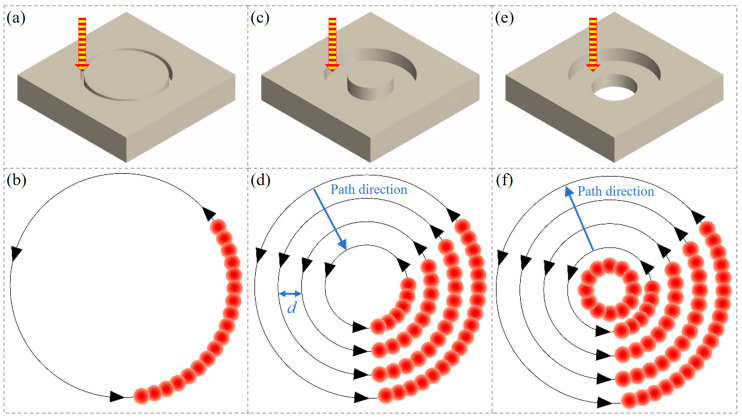
Water-jet-guided laser drilling strategies. (**a**) Single-pass annular cutting; (**b**) multi-pass annular cutting: from outside to inside; (**c**) multi-pass annular cutting: from inside to outside. (**d**) schematic diagram of motion trajectory in (**a**); (**e**) schematic diagram of motion trajectory in (**b**); (**f**) schematic diagram of motion trajectory in (**c**).

In the experiment, three water-jet-guided laser drilling strategies: single-pass annular cutting, multi-pass annular cutting (from outside to inside), and multi-pass annular cutting (from inside to outside). These techniques were utilized to fabricate micro-holes with a diameter of 0.8 mm, as depicted in Figure 2.

Single-pass annular cutting entails the precise manipulation of the workpiece’s motion trajectory via the regulation of the XY displacement worktable, resulting in the formation of a series of concentric circles with uniform diameters until the workpiece is fully penetrated, as shown in Figure 2a,b.

Multi-pass annular cutting pertains to a machining methodology wherein the scanning path assumes the form of concentric circles with varying radii, facilitating precise micro-hole fabrication through incremental feeding in a layer-by-layer manner. Specifically, when adopting an outside-to-inside scanning path, the water–light coupled energy beam initiates processing at the outermost circumference of the target aperture, subsequently shifting inward progressively layer by layer until the innermost circumference is fully processed, marking the conclusion of the first processing cycle. Subsequently, the processing path reverts back to the outermost circumference to commence the second processing cycle, with this cyclic process continuing iteratively until the entire machining operation is completed, as depicted in Figure 2c,d.

When adopting an inside-to-outside scanning path, the water-jet-guided laser coupling energy packet first processes an initial through-hole with a smaller diameter. Subsequently, the processing path expands outward layer by layer in the form of concentric circles until the first round of processing of concentric circles with a diameter of 0.8 mm is completed. Then, the processing path returns to the inner circle and repeats the aforementioned processing mode of expanding outward layer by layer, repeating this cycle until the micro-hole processing with a diameter of 0.8 mm is finally completed, as shown in Figure 2e,f.

The processing parameters for water-jet-guided laser drilling are shown in Table 3. Each set of process parameters was tested three times, and the results were averaged to reduce experimental error.

### 2.4. Experimental Characterization

After completing the micro-hole machining, the sample was placed in an anhydrous ethanol solution for 15 min of ultrasonic cleaning, aiming to remove the residue attached to the surface and inner wall of the micro-holes. Simultaneously, three evaluation indicators, namely micro-hole diameter, micro-hole roundness, and micro-hole taper, were used to evaluate the machining accuracy of the micro-holes.

**Figure 3:**
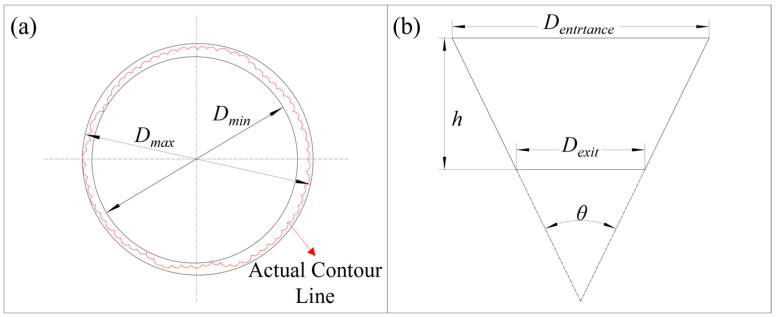
Evaluation of micro-hole machining quality. (**a**) Micro-hole roundness; (**b**) micro-hole taper.

Macroscopic observations of the surface morphology at the entrances and exits of micro-holes were performed using an ultra-depth-of-field microscope (DSX1000, Olympus, Tokyo, Japan), accompanied by measurements of the micro-hole diameters. The roundness of micro-holes pertains to the extent to which the machined surface approximates an ideal circle. The roundness can be measured using the minimum region method [33], as depicted in Figure 3a, with the calculation formula provided in Equation (1).

(1) $$H_{c}=\frac{D_{\min}}{D_{\max}}$$

In the formula, *D*_max_ represents the maximum hole diameter, *D*_min_ the minimum hole diameter, and *H_c_* the roundness of holes. A value of *H_c_* closer to 1 indicates that the morphology is closer to an ideal circle.

The micro-hole taper is used to evaluate the deviation between the entrance diameter and exit diameter. It can be calculated by measuring the average entrance diameter, average exit diameter, and hole depth of the micro-holes, as shown in Figure 3b, using Equation (2).

(2) $$\theta =\arctan\left(\frac{D_{entrance}-D_{exit}}{2h}\right)$$

In the formula, *θ* represents the conical angle of the micro-hole, *D_entrance_* denotes the average entrance diameter, *D_exit_* denotes the average exit diameter, and *h* represents the hole depth.

The microstructural morphology and distribution of alloy elements within micro-hole cross-sections were characterized using a field-emission scanning electron microscope (Merlin Compact, ZEISS, Oberkochen, Germany), equipped with an energy-dispersive spectrometer. Additionally, the surface roughness of the hole walls was assessed using a laser confocal microscope (KC-X1000, KathMatic, Nanjing, China), with the arithmetic mean deviation of the profile (Sa) employed as the metric for evaluating surface roughness.

## 3. Results and Discussion

### 3.1. Mechanism of Material Removal in Water-Jet-Guided Laser Drilling

**Figure 4:**
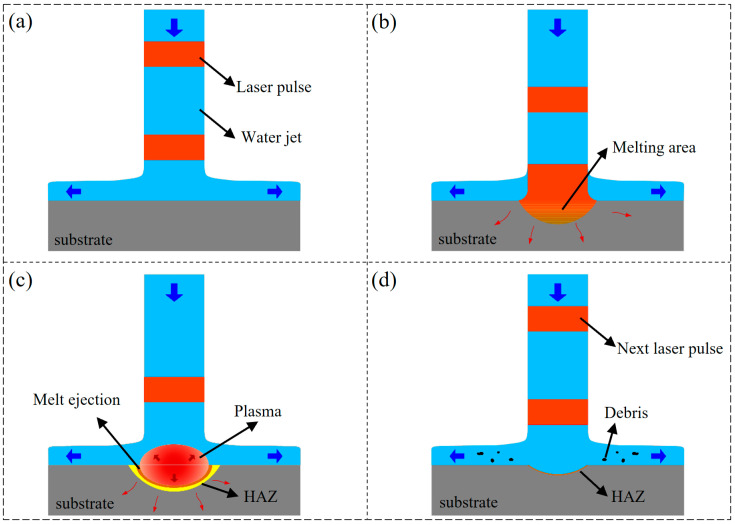
Material removal mechanism of water-jet-guided laser drilling. (**a**) Before pulsed laser action; (**b**) formation of molten pool and steam; (**c**) formation of plasma plume and shock wave; (**d**) after pulsed laser action.

In water-jet-guided laser technology, the laser source typically utilized is a nanosecond laser. The interaction between nanosecond pulsed lasers and materials primarily encompasses photothermal effects and photochemical reactions, with the fundamental process being the deposition of laser energy within the material. When the energy deposited in the material reaches a specific threshold, the material is removed via mechanisms such as melting, vaporization, and photochemical reactions, while simultaneously generating thermal effects and thermal damage at the periphery of the interaction zone. The core advantages of water-guided laser processing technology reside in its remarkable cooling effect and efficient material removal mechanism, both of which are attributed to the synergistic action of the water jet during the processing. Figure 4 demonstrates the material removal mechanism during the drilling of metal materials using water-jet-guided laser technology.

Initially, when a continuous water jet is applied to the material surface, the water medium uniformly diffuses radially from the jet’s impact point, encompassing the surrounding areas and forming an even, thin water layer on the upper surface of the workpiece. Throughout this process, the primary manifestation of the water jet’s effect on the material is the stable impact force generated upon the jet striking the object.

Secondly, upon reaching the material surface under the guidance of water jets, pulsed lasers with high power density cause the material within the interaction zone to absorb laser energy, subsequently inducing temperature elevation, melting, and mild vaporization. As the pulsed laser irradiation persists, a portion of the laser energy is absorbed by the vapor plume, undergoing ionization, and giving rise to a high-temperature and high-pressure plasma cloud in the region between the water jet and the material [20].

Subsequently, the plasma continuously absorbs a substantial amount of laser energy via the inverse bremsstrahlung mechanism, leading to a notable increase in both electron density and electron temperature per unit volume. This, in turn, propels the plasma to expand outwardly and generates impact pressure. At this juncture, the thin water layer serves as a confining layer, confining the plasma between itself and the material [34]. The synergistic effect of the plasma impact pressure and vapor pressure, both constrained by the thin water layer, accelerates the ejection process of the molten material. When the impact pressure generated by the plasma becomes sufficient to surpass the surface tension of the liquid metal and the gravitational pull of the liquid, it triggers explosive vaporization and evaporation, giving rise to high-magnitude pressure shock waves that effectively remove the residual melt layer within the processing area [21].

Eventually, upon cessation of the pulsed energy application, the plasma phenomena dissipate, prompting the water medium to subsequently flow into the cavity. This action concurrently scavenges heat and residual molten metal, thereby concluding a complete machining cycle. Furthermore, prior to the subsequent pulse interacting with the material, the water jet persists in cooling and flushing the machining area, restoring its temperature to levels close to ambient.

### 3.2. Effect of Drilling Strategy on the Morphology of Micro-Holes

**Figure 5:**
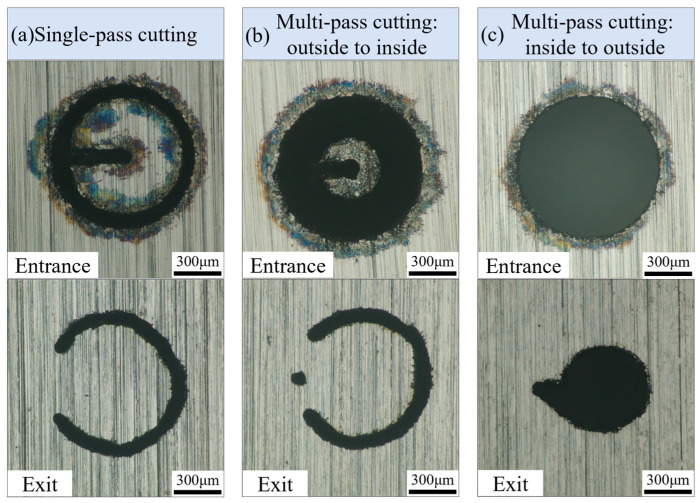
Effect of drilling strategy on micro-hole morphology. (**a**) Single-pass cutting; (**b**) multi-pass annular cutting from outside to inside; (**c**) multi-pass annular cutting from inside to outside.

The scanning speed of the water-jet-guided laser drilling is set to 25 mm/s, with a laser pulse frequency of 300 kHz, laser pulse width of 50 ns, and laser power of 180 W (single-pulse energy of 0.6 mJ). The effects of three drilling strategies on micro-hole morphology when the micro-holes remain in a non-through state are shown in Figure 5.

Figure 5a illustrates the surface morphology of micro-hole entrances and exits under the single-pass annular cutting strategy. Observations reveal a black annular ablation layer approximately 100 µm wide at the entrance edge, accompanied by significant deposition of molten resolidified debris. On the exit side, partial residual material remains and the hole circularity is compromised. The analysis indicates that molten droplets and vaporized products generated during the annular cutting process predominantly flow along the entrance surface. As the cutting groove deepens, the water accumulation at the groove bottom creates substantial resistance to the microjet impingement, leading to molten material resolidification and surface adhesion at the entrance edge. After initial micro-channel formation at the bottom, slag and water accumulation can only be expelled through the micro-hole exit, but at this stage the central column remains intact and the processing is incomplete. Continuous subsequent cutting operations are required until the central column finally separates, marking the completion of the micro-hole fabrication process.

Figure 5b illustrates the surface topography of the micro-hole inlet and outlet under the “outside-to-inside” multi-pass drilling strategy. The black oxide layer remains observable at the inlet edge, though the adhered molten material is significantly reduced. The outlet side exhibits residual material not fully removed, while the overall roundness of the micro-hole is notably enhanced. Analysis indicates that the strategy initially expels molten material from the inlet surface during machining, making complete avoidance of thermal oxidation and erosion at the inlet region unattainable. However, the multi-pass drilling effectively mitigates the counter-erosion effect caused by water accumulation in grooves, reducing the probability of molten material adhesion on the periphery of the hole to a certain extent, thereby improving the hole shape quality.

Figure 5c illustrates the surface morphology of the entrance and exit of micro-holes when employing the “inside-to-outside” multi-pass annular cutting water-jet-guided laser drilling strategy. The findings are as follows: (1) the amount of fusion residue adhering to the entrance edge is minimal, with a black oxide layer width of less than 50 µm; (2) the circularity of the micro-holes is notably superior to that achieved with other strategies, while the exit side exhibits smaller, circular apertures. Analysis of the underlying causes indicates that the water-jet-guided laser energy beam initially focuses on the central region of the micro-hole, achieving complete material removal of the central cylindrical area. Subsequently, the energy beam expands radially, scanning in successive annular passes and gradually increasing the exit aperture size. Since a continuous exit channel has formed at the bottom of the bore, molten residues and cooling water can be promptly evacuated, significantly mitigating thermal effects and the risk of redeposition. No significant thermal damage or fusion residue is observed on the upper surface of the material. In summary, this strategy offers advantages such as high surface quality, a small taper angle, and excellent circularity, with complete material removal within the micro-holes. Consequently, the “inside-to-outside” multi-pass annular cutting water-jet-guided laser drilling strategy is uniformly adopted for all subsequent experiments.

### 3.3. The Effect of Single-Pulse Energy on the Machining Precision of Micro-Holes

**Figure 6:**
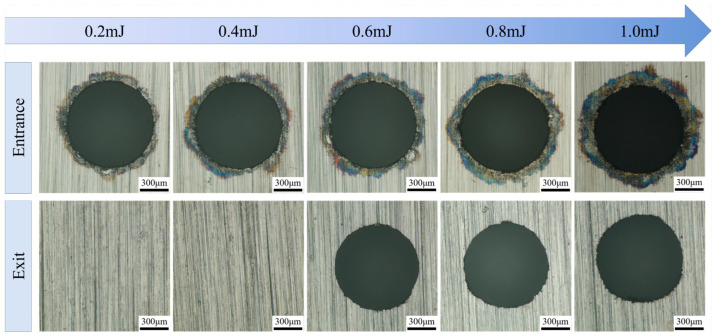
Effect of single-pulse energy on micro-hole surface morphology.

To explore the influence of single-pulse energy on the accuracy of micro-hole drilling in water-jet-guided laser processing, experiments were conducted under constant process parameters: the scanning speed of the coupled energy beam was set at 20 mm/s, with a laser pulse frequency of 300 kHz and a laser pulse width of 50 ns. Single-pulse energy was utilized as the independent variable, with five gradient levels selected: 0.2 mJ, 0.4 mJ, 0.6 mJ, 0.8 mJ, and 1.0 mJ, corresponding to laser powers of 60 W, 120 W, 180 W, 240 W, and 300 W, respectively. Figure 6 illustrates the surface morphology of the micro-hole entrances and exits under varying single-pulse energy conditions. The experimental results revealed that when the single-pulse energy was less than 0.4 mJ, material removal was inadequate, preventing the micro-holes from fully penetrating. Notably, distinct ring-shaped black areas emerged at the entrance edges, with their areas monotonically increasing as the single-pulse energy rose. Conversely, no similar black rings were observed at the exit edges. Analysis indicates that upon contact with the workpiece surface, the water-jet-guided energy beam formed a radial thin water film. During laser propagation at the substrate–water film interface, scattering and refraction effects ensued, resulting in localized energy redistribution. This redistribution induced micro-scale ablation and oxidation reactions in the surrounding areas of the holes, ultimately leading to the formation of the observed black ring-shaped morphology.

**Figure 7:**
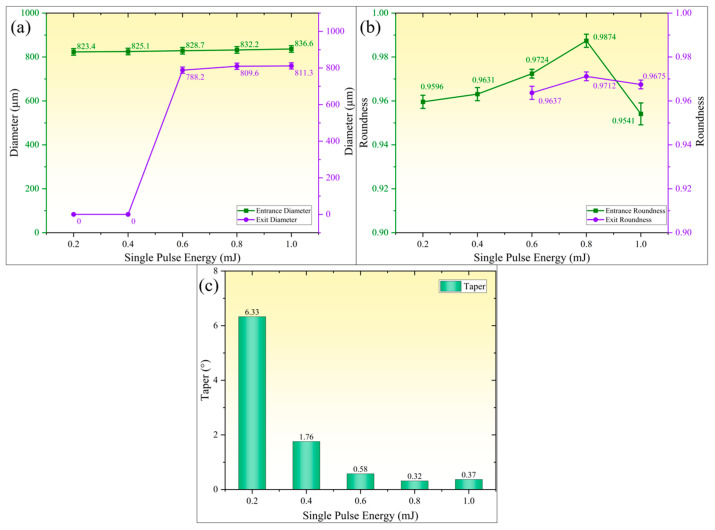
Effect of single-pulse energy on micro-hole accuracy. (**a**) Entrance and exit diameters; (**b**) entrance and exit roundness; (**c**) micro-hole taper.

Figure 7 reveals the influence patterns of single-pulse energy on geometric precision parameters of micro-holes (hole diameter, roundness, and taper). As shown in Figure 7a, both entrance and exit diameters of micro-holes exhibit monotonic growth with increasing single-pulse energy, though their rate of change shows significant disparity. When single-pulse energy increases from 0.2 mJ to 1.0 mJ, entrance diameter gradually expands from 823.4 µm to 836.6 µm (1.5% increase), demonstrating a relatively gradual expansion trend. However, when single-pulse energy is below 0.4 mJ, insufficient material removal results in incomplete micro-hole formation. Upon energy escalation from 0.6 mJ to 1.0 mJ, exit diameter rapidly increases from 798.2 µm to 811.3 µm followed by saturation, indicating a more sensitive threshold effect of energy density on outlet expansion. Figure 7b demonstrates the variation in micro-hole roundness with single-pulse energy. For energies below 0.4 mJ, micro-holes remain in blind hole state with maximum entrance roundness of 0.9631. As energy increases, roundness continuously improves, reaching peak values at 0.8 mJ (entrance roundness 0.9874, exit roundness 0.9852). Further energy increment causes fusion residue adhesion and chipping defects at entrance edges, resulting in roundness decline to 0.9541. The outlet roundness remains relatively stable due to the cooling effect of the water jet, showing insignificant variation. Figure 7c illustrates the variation in micro-hole taper with single-pulse energy. For non-through micro-holes, the initial taper was measured as 6.33° using axial cross-sectional sectioning method; with increasing single-pulse energy, the taper rapidly decreased to 0.32° (0.8 mJ), indicating that high energy density effectively suppressed wall tilting. However, when energy was further increased to 1.0 mJ, the taper mildly increased to 0.37°, which is attributed to the expansion of the secondary heat-affected zone and melting–redeposition at the hole entrance caused by higher energy levels.

Studies have shown that when the single-pulse energy is relatively low (0.2 mJ and 0.4 mJ), the ablation process primarily manifests as thermal evaporation and melt migration, with relatively lower material removal rates and inefficient melt ejection. Particularly at the exit end, residual un-removed material results in poor roundness and taper of micro-holes. As the single-pulse energy increases, the vapor plume becomes ionized and initiates a liquid-phase explosion, generating an instantaneous high-pressure shock wave that significantly enhances effective material removal. Simultaneously, the enhanced recoil pressure improves melt ejection efficiency, promoting continuous forward expansion of micro-holes from their bottoms while enlarging exit diameters and substantially reducing taper. However, when the single-pulse energy becomes excessively high, the plasma plume shielding effect and scattering–refraction interference of gaseous products significantly reduce laser energy transmission efficiency to the hole bottom, leading to decreased material removal rate at the bottom. Consequently, the expansion rate of exit diameters becomes smaller than that of entrance diameters, resulting in a slight rebound in taper.

### 3.4. The Effect of Scanning Speed on the Machining Precision of Micro-Holes

**Figure 8:**
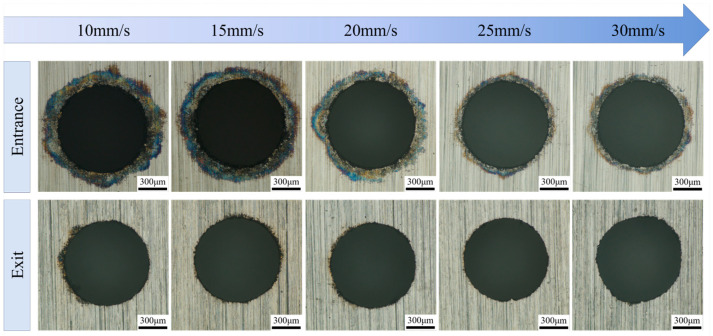
Effect of scanning speed on micro-hole surface morphology.

To explore the impact of scanning speed on the precision of micro-hole machining in water-jet-guided laser processing, experiments were conducted with the following laser parameters: a fixed laser power of 240 W (corresponding to a single-pulse energy of 0.8 mJ), a pulse width of 50 ns, and a pulse frequency of 300 kHz. The scanning speed of the water-jet-guided laser beam served as the sole variable, with values set at 10 mm/s, 15 mm/s, 20 mm/s, 25 mm/s, and 30 mm/s, respectively. Figure 8 illustrates the surface morphology characteristics of the micro-hole entrances and exits at various scanning speeds. A consistent observation was the presence of dark circular areas at the entrance edges, with their area notably expanding as the scanning speed decreased. Notably, when the scanning speed surpassed 25 mm/s, there was a marked improvement in the integrity of the entrance edges, accompanied by a continuous enhancement in surface quality. Conversely, at scanning speeds below 15 mm/s, the entrance edges exhibited accumulation of molten material along with minor edge chipping defects. In stark contrast, no apparent heat-affected zones or ablation marks were observed at the exit edges, suggesting that the influence of scanning speed on exit morphology is substantially weaker compared to its effect on entrance characteristics.

**Figure 9:**
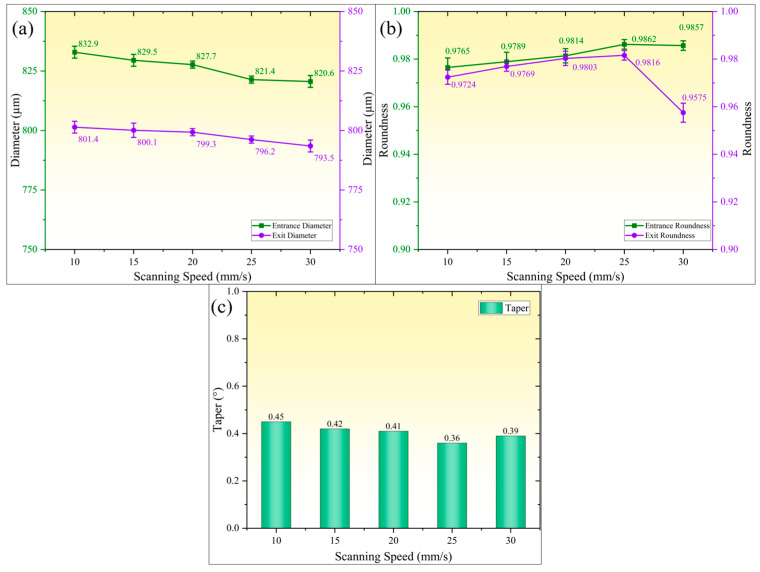
Effect of scanning speed on micro-hole accuracy. (**a**) Entrance and exit diameters; (**b**) entrance and exit roundness; (**c**) micro-hole taper.

Figure 9 illustrates the influence pattern of scanning speed on geometric accuracy parameters of micro-holes (hole diameter, roundness, and taper). As shown in Figure 9a, when the scanning speed increases from 10 mm/s to 30 mm/s, the entrance and exit diameters of micro-holes exhibit synchronized and gradual reduction. Specifically, the entrance diameter decreases from 832.9 µm to 820.6 µm, while the exit diameter reduces from 801.4 µm to 793.5 µm, with variations in less than 1.5% in both cases. This phenomenon can be attributed to the following mechanisms. At lower scanning speeds, higher laser energy input per unit time and area causes minor thermal damage to the entrance edge despite effective suppression of heat-affected zone expansion through microjet scouring effects. As scanning speed increases, the laser energy density per unit area gradually decreases while the interaction time between microjet and workpiece surface shortens. These dual effects synergistically inhibit molten material deposition and thermal zone expansion, thereby achieving synchronized reduction in both entrance and exit diameters.

Figure 9b illustrates the variation in the roundness of micro-holes with scanning speed. Both the entrance and exit roundness of micro-holes exhibit an increasing–decreasing trend, albeit with notable differences in fluctuation amplitudes. When the scanning speed increased from 10 mm/s to 25 mm/s, the entrance roundness rose from 0.9765 to 0.9862, while the exit roundness increased from 0.9724 to 0.9816. The significant improvement in entrance roundness is attributed to the presence of minor thermal ablation and trace adhered molten material at the entrance edge under low-speed conditions. As the speed increased, the reduction in the heat-affected zone and melt deposition quantity smoothed the micro-hole edges, thereby enhancing the roundness. However, when the scanning speed further escalated to 30 mm/s, the exit roundness exhibited abrupt fluctuations and abruptly decreased to 0.9575. This decline is caused by the increased likelihood of water jet disruption-induced rupture at higher speeds, resulting in laser scattering and compromising the continuity of the exit processing. Simultaneously, the reduced spot overlap rate led to unstable laser energy input per unit area, causing significant energy absorption fluctuations in the material and consequently lowering the roundness of the micro-hole exit.

Figure 9c illustrates the variation patterns of the micro-hole taper with scanning speed. When the scanning speed increases from 10 mm/s to 25 mm/s, the taper monotonically decreases from 0.45° to 0.36°. Further elevation to 30 mm/s results in a reversal of the taper to 0.39°. This phenomenon is attributed to the enhanced suppression of micro-erosion at the hole wall edges and improved geometric accuracy of the hole shape, which continuously reduces the taper. However, when the scanning speed surpasses the critical threshold, the reduced effective pulse density per unit area leads to insufficient total incident energy, resulting in incomplete material removal and subsequent taper increase. Additionally, excessively low scanning speeds significantly compromise the efficiency of water-jet-guided laser machining, failing to meet large-scale production requirements. Therefore, during practical process optimization, a balanced compromise between machining quality and efficiency must be achieved by constraining the scanning speed within a rational range that simultaneously ensures hole shape precision and processing efficiency.

### 3.5. The Effect of Laser Pulse Frequency on the Machining Precision of Micro-Holes

**Figure 10:**
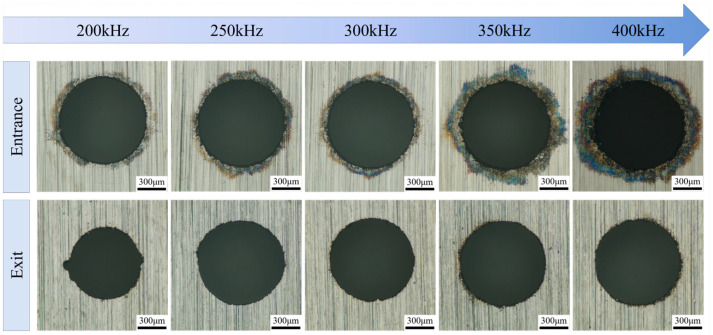
Effect of laser pulse frequency on micro-hole surface morphology.

To explore the impact of pulse frequency in water-jet-guided laser processing on the accuracy of micro-hole fabrication, experiments were conducted with the following laser process parameters: a single-pulse energy of 0.8 mJ, a pulse width of 50 ns, and a scanning speed of 25 mm/s. The pulse frequency was varied as the sole independent variable, with settings of 200 kHz, 250 kHz, 300 kHz, 350 kHz, and 400 kHz, corresponding to average laser powers of 160 W, 200 W, 240 W, 280 W, and 320 W, respectively. Figure 10 illustrates the surface morphologies of micro-hole entrances and exits under various pulse frequency conditions. The results indicate that the influence pattern of laser pulse frequency on micro-hole morphology is largely consistent with that observed for variations in single-pulse energy. At a pulse frequency of 200 kHz (laser power of 160 W), material removal on the exit side is significantly insufficient, resulting in rough micro-hole edges. As the pulse frequency increased, accompanied by a corresponding increase in laser power, the thermal ablation effect on the material became markedly enhanced. When the pulse frequency reached 400 kHz (laser power of 320 W), the black ablation area at the entrance edge expanded significantly, with molten residues adhering to the perimeter of the hole mouth.

**Figure 11:**
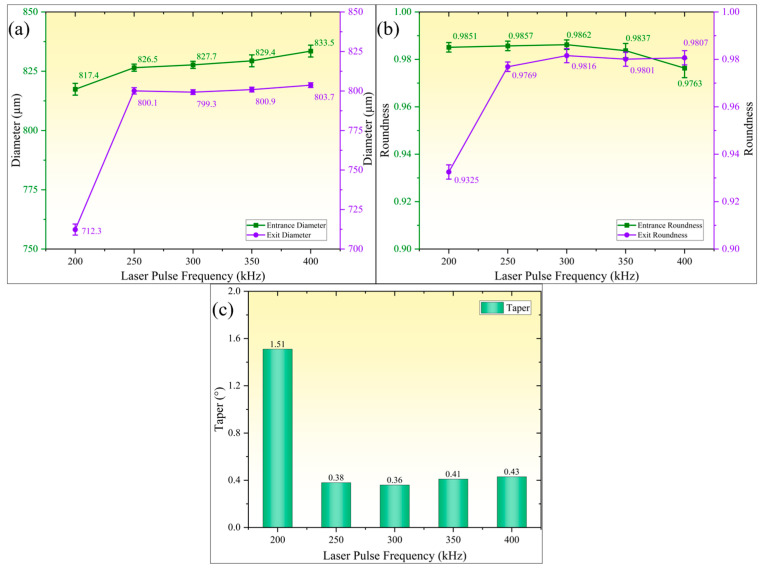
Effect of laser pulse frequency on micro-hole accuracy. (**a**) Entrance and exit diameters; (**b**) entrance and exit roundness; (**c**) micro-hole taper.

Figure 11 illustrates the influence patterns of pulse frequency on geometric accuracy parameters of micro-holes (hole diameter, roundness, and taper). As shown in Figure 11a, both entrance and exit diameters of micro-holes exhibit monotonic increases with rising pulse frequency. When pulse frequency increased from 200 kHz to 400 kHz, entrance diameter expanded from 817.4 µm to 833.5 µm, while exit diameter increased from 712.3 µm to 803.7 µm. Figure 11b reveals non-monotonic variations in micro-hole roundness. The entrance roundness remained stable before decreasing, while the exit roundness initially increased and then stabilized. When pulse frequency rose from 200 kHz to 300 kHz, entrance roundness maintained within 0.9851–0.9862 range, while exit roundness improved from 0.9325 to 0.9816. Further increasing pulse frequency to 400 kHz caused entrance roundness to decrease gradually to 0.9763, whereas exit roundness remained above 0.98 and stabilized. Figure 11c demonstrates characteristics of taper decreasing first and then slightly rebounding with increasing pulse frequency. At 200 kHz, maximum taper reached 1.51°, which continuously decreased to minimum 0.36° at 300 kHz. When pulse frequency further increased to 400 kHz, taper slightly increased to 0.43°. Under constant single-pulse energy conditions, higher pulse frequency implies increased pulse number per unit time, resulting in greater cumulative laser energy per unit area and more pronounced material removal effects. However, exceeding critical threshold of accumulated laser energy intensifies thermal damage, consequently decreasing micro-hole roundness. Conversely, insufficient energy accumulation at low pulse frequencies leads to inadequate material removal, resulting in increased taper and reduced roundness.

Further analysis reveals that the role of pulse frequency in water-jet-guided laser drilling primarily manifests as the competitive coupling between the “heat accumulation effect” and the “plasma shielding effect” [35,36]. At high laser pulse frequencies, the thermal effects from preceding pulses have not fully dissipated before subsequent pulses interact with the material, enhancing the heat accumulation effect through increased material absorption. Concurrently, shortened pulse intervals prevent complete dissipation of plasma generated by preceding pulses, resulting in significant plasma shielding effects where subsequent pulse energy is predominantly absorbed, reflected, and refracted. This leads to reduced laser energy utilization efficiency. Under low-frequency conditions, prolonged pulse intervals substantially reduce plasma concentration, effectively suppressing shielding effects and enabling efficient energy deposition into the material. Thus, both effects intensify with frequency elevation, yet exhibit mutual constraints: the former enhances energy coupling efficiency while the latter diminishes energy transmission. Optimal pulse frequency selection allows simultaneous maximization of heat accumulation effects and controlled suppression of plasma shielding effects, achieving efficient low-loss micro-hole processing.

## 4. Optimization Verification and Analysis

### 4.1. Micro-Morphological Analysis of Micro-Hole Aperture

**Figure 12:**
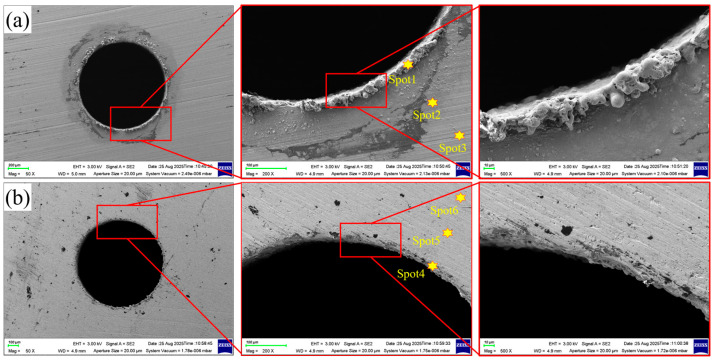
Microscopic morphology of micro-hole entrance and exit. (**a**) Micro-hole entrance; (**b**) micro-hole exit.

Based on the aforementioned experimental results, micro-holes were produced utilizing the water-jet-guided laser multi-pass ring-cutting technique, employing the optimal process parameters: a single-pulse energy of 0.8 mJ, a scanning speed of 25 mm/s, a pulse frequency of 300 kHz, and a pulse width of 50 ns. Upon averaging multiple measurements, the micro-holes exhibited an entrance diameter of 822.7 µm with a roundness of 0.9893, and an exit diameter of 800.5 µm with a roundness of 0.9865, accompanied by a minimal cone angle of only 0.32°. The processing time for each hole was approximately 60 s. The scanning electron microscopy (SEM) images presented in Figure 12 reveal that the surface quality of both the entrance and exit of the micro-holes is excellent, devoid of any noticeable cracks, recast layers, or heat-affected zones. A slight amount of molten material adheres to the rim of the entrance, while sputtering ablation marks are discernible around the perimeter. Conversely, the exit side displays no apparent molten material or ablation marks, albeit the hole edge exhibits a subtle serrated pattern.

**Figure 13:**
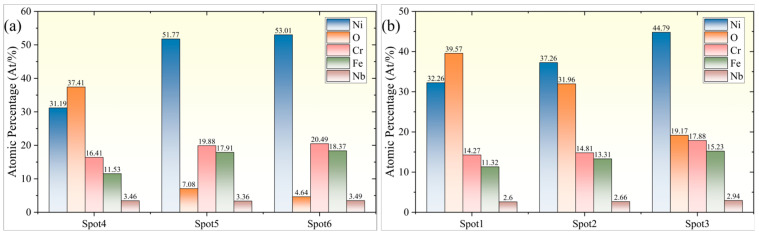
Element distribution at the micro-hole entrance and exit. (**a**) Micro-hole entrance; (**b**) micro-hole exit.

To further elucidate the changes in chemical composition on the micropore surface, energy-dispersive spectroscopy (EDS) was employed to analyze the elemental distribution on the micropore inlet and outlet surfaces (with data normalization processing). As shown in Figure 12, three points, namely Spot1, Spot2, and Spot3, were selected radially from the edge of the micropore inlet side outwards. Similarly, three points, namely Spot4, Spot5, and Spot6, were selected on the outlet side. The results are presented in Figure 13. It was observed that the atomic fraction of Ni on the inlet side increased sharply from 32.66% at Spot1 to 44.79% at Spot3. Correspondingly, the atomic fraction of O decreased slightly from 39.57% to 31.96%, and then significantly dropped to 19.17%. The atomic fraction of Cr gradually increased from 14.72% to 17.88%, with a change of approximately 25.3%. The atomic fraction of Fe increased from 11.32% to 15.23%, representing a change of over 34.5%. On the micro-hole outlet side, the atomic fraction of Ni increased sharply from 31.19% at Spot4 to 51.77% at Spot5, and then slightly increased to 53.01% at Spot6. The atomic fraction of O decreased sharply from 37.41% to 7.08%, and then slightly decreased to 4.64%. Meanwhile, the atomic fractions of both Cr and Fe showed significant increases.

The distribution characteristics of the aforementioned elements are highly consistent with the macroscopic morphological features of the micro-hole entrance and exit. During the water-jet-guided laser drilling process, material removal is primarily attributed to the laser-induced photothermal effect. The material in the region affected by the water–light coupled energy packet melts, forming a recast layer around the pore wall, leading to a significant increase in the oxygen (O) element content at the pore entrances, namely Spot1 and Spot3. Compared to Spot3, which is farther from the entrance, Spot2, located immediately adjacent to the entrance, exhibits more pronounced elemental fluctuations, indicating the deposition of ablation products in the Spot2 region, while the Spot3 region is characterized as a thermally oxidized affected area. The elemental content changes at Spot5 and Spot6 on the exit side are relatively smooth, indicating a weaker thermal impact effect in this region and no distinct thermally affected zone formed.

### 4.2. Micro-Morphological Analysis of Micro-Hole Inner Walls

**Figure 14:**
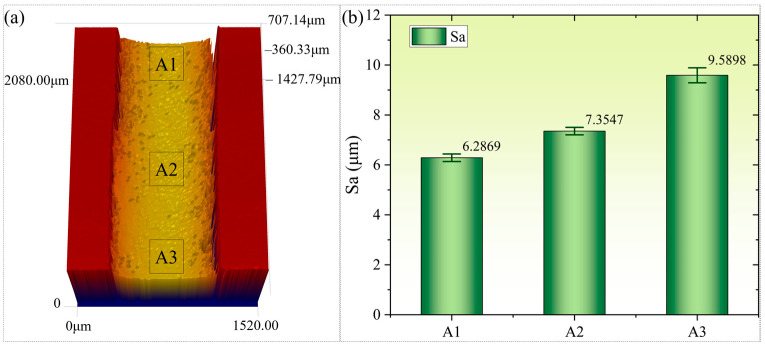
Micro-hole wall morphology and roughness. (**a**) Hole wall morphology; (**b**) hole wall roughness.

Figure 14 shows the 3D morphology of the micro-hole inner wall and the roughness of the hole wall, measured by a confocal laser scanning microscope after slicing the micro-hole along its diameter. As can be seen from Figure 14a, the contour of the micro-hole inner wall remains consistent from the entrance section (A1), the middle section (A2), to the exit section (A3), with no significant changes. Areas of 100 μm×100 μm were selected in the three sections to measure the roughness Sa of the hole wall, and the results are shown in Figure 14b. The roughness of the hole wall exhibits slight variations along the axial direction, with the highest Sa value of 9.5898 μm in the exit section. This phenomenon can be attributed to the continuous disturbance of the water jet near the hole wall by reverse splashing droplets and slag during the water-jet-guided laser drilling process. As the processing depth increases, the disturbance gradually intensifies, making it difficult for the fine water beam to maintain an ideal cylindrical shape, resulting in significantly worse roughness in the exit area compared to the entrance area. The higher roughness of the gas film hole wall can reduce the heat transfer efficiency of the cooling gas. Further optimization of processing strategies and process parameters is needed to meet the application requirements of high-performance gas film holes.

**Figure 15:**
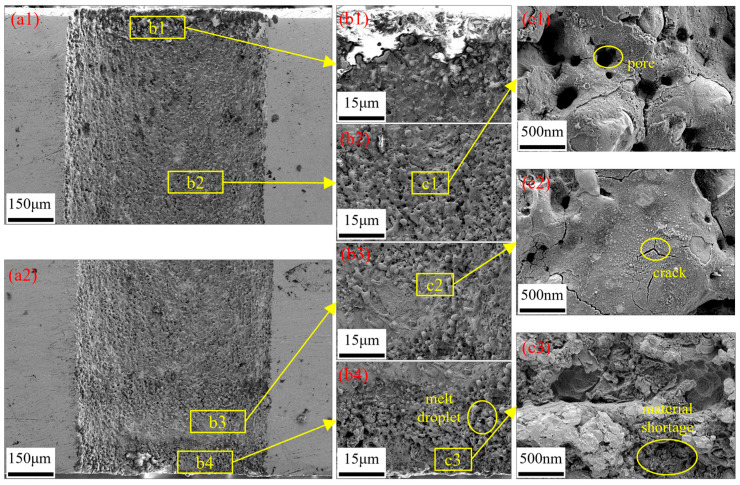
Micro-hole wall microtopography. (**a1**) Micro-hole wall topography near the entrance; (**a2**) micro-hole wall topography near the exit; (**b1**–**b4**,**c1**–**c3**) magnified views of specific areas.

As shown in Figure 15, by analyzing the cross-sectional morphological characteristics of micro-holes processed by water-jet-guided laser, the hole wall surface can be divided into four regions based on morphological features: resolidified zone, bulged zone, concave zone, and fractured zone. The resolidified zone (Figure 15(b1)) is concentrated at the entrance of the micro-hole. The edge contour of this zone exhibits a smooth transition morphology, accompanied by random attachment of micron-sized metal droplets. Its formation mechanism can be attributed to the transient thermal effect generated during the interaction between the laser and the metal material, leading to phase transformation, melting, and even local vaporization of the material. After the laser action terminates, the molten material undergoes a rapid solidification process, resulting in the formation of a resolidified zone with specific morphological characteristics at the edge of the hole entrance.

The bulged zone (Figure 15(b2)) and concave zone (Figure 15(b3)) are distributed almost throughout the entire micro-holes wall surface. The bulged zone exhibit significant undulations and are densely covered with small-scale pores with diameters less than 1 µm. Their formation is attributed to the melting or vaporization of materials induced by instantaneous high temperatures during water-jet-guided laser processing. When metal vapor undergoes gaseous fracture, a micro-explosion effect occurs, resulting in hole defects on the surface [37]. Additionally, multiple cycles are employed during the water-jet-guided laser drilling process. The materials in the bulged zone undergo repeated melting, cooling, and evaporation processes, leading to the formation of this micro-morphology with a dense distribution of pores. The morphology of the concave zone is mainly composed of block-like platforms, with microcracks distributed across the surface. These cracks divide the overall structure into micro-blocks of varying sizes and shapes. The edges of the blocks are densely populated with submicron-scale pores, resulting in significant surface undulations. This phenomenon occurs because after the pulsed laser is coupled to the workpiece surface through a high-pressure water jet, energy accumulates in local areas, causing material melting and evaporation. Most of the molten metal is removed by the high-speed water jet. However, due to the limited erosion capacity of the water jet, a small amount of molten metal re-solidifies on the cutting surface, forming a remelted layer in the concave zone. Due to the rapid heating and cooling characteristics of water-jet-guided laser processing, significant thermal stress is generated within the remelted layer. When the stress exceeds its yield strength, cracks initiate [38], eventually evolving into a surface structure coexisting with block-like platforms and cracks.

The fractured zone (Figure 15(b4)) is concentrated at the micro-hole exit. The surface of the fractured zone is distributed with spherical droplets and a small number of large-scale pores. At the bottom of the fractured zone, blocky platforms can be observed, but their edges are significantly undulating, and there is a phenomenon of material loss. The formation of this morphology is attributed to the strong scouring effect of high-pressure water jets. On the one hand, molten metal is thrown away from the matrix, leaving behind large-scale pores; on the other hand, the ejected metal is rapidly cooled by the water jet and solidifies into spherical droplets under the action of surface tension. In summary, blocky platforms, spherical droplets, and large-scale pores together constitute the micro-morphology of the fractured zone.

## 5. Conclusions

This study explores the application of a 1064 nm wavelength water-jet-guided laser for ring-cutting hole drilling in Inconel 718. It elucidates the influence mechanisms of crucial process parameters, such as single-pulse energy, scanning speed, and pulse frequency, on the morphology and geometric accuracy of micro-holes. Based on these findings, an optimal approach for achieving high-efficiency and high-precision hole drilling is identified. The primary conclusions are summarized below:

(1) The multi-pass annular water-jet-guided laser drilling strategy from inside to outside can enhance the erosion effect of water jet on molten material, reduce the heat-affected area and residual molten material on the entrance surface of micro-hole.
(2) When processing micro-holes in Inconel 718 using a water-jet-guided laser at 1064 nm wavelength, the optimal process parameter combination is single-pulse energy of 0.8 mJ, scanning speed of 20 mm/s, and laser pulse frequency of 300 kHz. Under this parameter configuration, high-quality micro-holes with an entrance diameter of 822.7 µm, roundness of 0.9893, taper of 0.32°, and surface roughness Sa less than 9.58 µm can be fabricated.
(3) Based on the cross-sectional morphology characteristics of micro-holes processed by water-jet-guided laser machining, the surfaces of micro-holes can be divided into four distinct regions: resolidified zone, bulged zone, concave zone, and fractured zone. The resolidified zone and fractured zone represent unique morphologies at the entrance and exit positions of micro-holes, respectively. The bulged zone and concave zone are distributed throughout the micro-hole walls, with their formation mechanisms being closely related to the photothermal effects and sudden heating and rapid cooling characteristics inherent in the water-jet-guided laser machining process.
(4) Through the observation of the entrance, exit, and wall profiles of micro-holes, it was discovered that water-jet-guided laser processing exhibits exceptional performance in minimizing recast layers and heat-affected zones, as well as maintaining bore wall cleanliness. This technique effectively mitigates the thermal effects and oxidation damage associated with traditional long-pulse laser processing, thereby facilitating high-quality and efficient machining of Inconel 718.

## Figures and Tables

**Table 1:** Main chemical composition of Inconel 718.

Element	Ni	Cr	Fe	Nb	Mo	Ti	Al
**Content, wt%**	50~55	17~21	Bal.	4.75~5.5	2.8~3.3	0.9~1.2	0.2~0.8

**Table 2:** Mechanical properties of Inconel 718.

Values	Hardness (HB)	Modulus (GPa)	Tensile Strength (MPa)	Yield Stress (MPa)
Inconel 718	363	206	1200	1030

**Table 3:** Process parameters for water-jet-guided laser drilling.

Parameter	Value
Single-pulse energy/(mJ)	0.2, 0.4, 0.6, 0.8, 1.0
Processing speed/(mm/s)	10, 15, 20, 25, 30
Pulse frequency/(kHz)	200, 250, 300, 350, 400
Number of cycles	20
Wavelength/nm	1064
Water pressure/MPa	35
Water-jet diameter/µm	60
Flow rate/NL·min^−1^	0.5
Nozzle-to-workpiece distance/mm	70
Beam spot diameter/µm	15
Nozzle incident angle/°	90
Beam focal position/mm	100.3

## Data Availability

The original contributions presented in this study are included in the article. Further inquiries can be directed to the corresponding author.
